# Usefulness of lung ultrasound B-lines in connective tissue disease-associated interstitial lung disease: a literature review

**DOI:** 10.1186/s13075-017-1409-7

**Published:** 2017-09-18

**Authors:** YuKai Wang, Luna Gargani, Tatiana Barskova, Dan E. Furst, Marco Matucci Cerinic

**Affiliations:** 1 Department of Experimental and Clinical Medicine, University of Florence, Florence, Italy; 2 Department of Geriatric Medicine, Division of Rheumatology AOUC, University of Florence, Florence, Italy; 3 Department of Rheumatology and Immunology, Shantou Central Hospital, No 114 Waima Road, Shantou, 515041 Guangdong China; 4 Institute of Clinical Physiology, National Research Council, Via Moruzzi 1, 56124 Pisa, Italy; 5 Division of Rheumatology, Department of Medicine, University of California at Los Angeles, Los Angeles, CA USA

**Keywords:** Lung ultrasound, B-lines, Pleural irregularity, High-resolution computed tomography, Connective tissue diseases, Interstitial lung disease, Systemic sclerosis, Rheumatoid arthritis, Sjögren’s syndrome, Anti-synthetase syndrome

## Abstract

Interstitial lung disease (ILD) is a major pulmonary manifestation of connective tissue disease (CTD), leading to significant morbidity and mortality. Chest high-resolution computed tomography (HRCT) is presently considered the diagnostic gold standard for pulmonary fibrosis diagnosis and quantification in the clinical arena. However, not negligible doses of ionizing radiation limit the use of HRCT, especially for serial follow-up in younger female patients. In the past decade, lung ultrasound (LUS) has been proposed to assess ILD by detecting and quantifying sonographic B-lines. Previous studies demonstrate that B-lines have a good diagnostic accuracy, especially high sensitivity, and correlate well with HRCT findings, suggesting LUS as a novel, non-invasive, and non-ionizing imaging method to be used in patients with CTD-ILD. Although preliminary data are promising, challenges and controversies still remain. For example, the mechanisms of B-line generation are not fully understood; the diagnostic accuracy and performance characteristics of LUS partially depend on the scanning scheme and scoring system used; and up-to-date B-lines cannot discriminate the early cellular inflammation from the chronic fibrotic phase in CTD-ILD. Therefore it is important for clinicians to understand the strengths and limitations of LUS in CTD-ILD patients, to maximize its value.

## Background

The lung parenchyma has always been considered a “forbidden zone” for ultrasound (US), because air is not a favourable medium for transmission of US waves. As a consequence, thoracic US was originally limited to the study of superficial pleural conditions, such as tumours, effusions, and to guide invasive procedures [1]. In the last decade, the so-called B-lines have been recognized as the sonographic sign of the pulmonary interstitial syndrome [2]. This sign is thought, at present, to reflect partial deaeration of the lung, which may be due to fluid accumulation [1–4] or deposition of collagen tissue [1, 2]. Studies in patients with diffuse parenchymal lung diseases have attempted to highlight the usefulness of LUS as a complementary modality to traditional radiologic imaging [5, 6]. In this paper, we review all data about the usefulness of B-lines to CTD-ILD, underlining the strengths and limitations.

## What are B-lines?

In radiologic imaging, the term “artefact” describes any part of an image which does not accurately represent the anatomic structures present within the subject being evaluated [7]. B-lines are defined as discrete laser-like vertical hyperechoic reverberation artefacts that arise from the pleural line, extend to the bottom of the screen without fading, and move synchronously with respiration [8]. B-lines are visible when the lung parenchyma air content is partially decreased and/or the interstitial space is volumetrically expanded, such as in pulmonary oedema of various aetiologies and interstitial lung disease [9–11]. It is important to underline that B-lines cannot be clearly correlated to a specific anatomical structure, but rather they are correlated to the changes in the physical properties of the lung [10]. Soldati et al. [9–11] hypothesized that the mechanism underlying B-line formation is reverberation coherent with topologic and pathologic variations of the lung interstitium. Although the results of these first experiments are promising, the biophysics and exact genesis of B-lines are not yet fully elucidated.

## B-lines in connective tissue disease-associated interstitial lung disease

Pulmonary involvement is a significant cause of CTD-related morbidity and mortality [12, 13]. Interstitial lung disease is a frequent parenchymal manifestation of CTDs. Although the pathogenesis of CTD-ILD is varied and not yet fully understood, early detection and therapy may improve the prognosis [14]. To date high-resolution computed tomography (HRCT) is the gold standard to diagnose CTD-ILD [15, 16]. Unfortunately, HRCT cannot be repeated very often because it has a high cost and is associated with high radiation exposure [17, 18]. Furthermore, although pulmonary function tests (PFTs) are valuable in screening and following-up for CTD-ILD, they are not always impaired in early stages and may not reflect the degree of fibrosis [19]. Lung biopsy is a powerful tool to establish a definite histopathologic diagnosis of ILD, but the invasiveness and the possibility of sampling errors limit its clinical application [20]. Since LUS is a non-invasive and non-ionizing modality, rheumatologists and internists attempted to use it to assess the presence of CTD-ILD. Preliminary data showed that the number of B-lines had a good correlation with the HRCT fibrosis pattern, and good diagnostic accuracy, especially sensitivity [21], expanding the armamentarium for diagnosis and follow-up of CTD-ILD.

## B-lines in systemic sclerosis-associated interstitial lung disease

Systemic sclerosis (SSc) is most often associated with ILD, with up to 90% of patients exhibiting evidence of ILD on HRCT [22]. Accordingly, the majority of studies about B-lines in CTD-ILD focused on SSc. The use of B-lines has been partially validated in SSc-ILD. Different studies, as discussed in the following, established the use of B-lines in over 400 patients with a wide range of disease duration, type, and severity.

A scoring system for LUS is needed to examine construct and criterion validity. In the past decade, some scoring methods have been developed and used to quantify the disease. In the first study about B-lines in SSc-ILD, a B-line score was calculated in 33 SSc patients by summing the total number of B-lines on the anterior and posterior chest. A total of 72 scanning sites were analysed. The examination was considered positive when the B-line sum in all scanning sites was > 10 [23]. In another study, a positive examination was defined either when ≥ 3 B-lines were present in at least two adjacent scanning sites or when a total of > 5 B-lines was recorded [24]. Gutierrez et al. [25] compared two different LUS methods to assess the CTD-ILD in a single cohort of patients (Table 1). The number of B-lines in 50 scanning sites (comprehensive assessment) and 14 scanning sites (simplified assessment) located among the bilateral anterior, medial, and posterior chest was counted. For the comprehensive assessment a B-line semi-quantitative score was defined by 0 = normal (<10 B-lines), 1 = mild (11–20 B-lines), 2 = moderate (21–50 B-lines), and 3 = marked (>50 B-lines), whereas for the simplified assessment the semi-quantitative score was 0 = normal (<5 B-lines), 1 = mild (6–15 B-lines), 2 = moderate (16–30 B-lines), and 3 = marked (>30 B-lines). The study found a significant correlation between the two scoring systems (*p* = 0.0001), with κ values for the inter-observer simplified LUS assessment in the range of 0.769–0.885, and concordance correlation coefficient values for the intra-observer reliability from 0.856 to 0.955. The simplified method required less time than the comprehensive examination (mean 8.6 ± 1.4 minutes vs 23.3 ± 4.5 minutes, *p* < 0.0001). Recently, fewer scanning sites (only 10) were evaluated based on the prevalence of B-line distribution. Results found that this modified scoring system had a good correlation with HRCT (correlation coefficient = 0.695, *p* < 0.001), good intra-observer reliability (κ value = 0.838), and was the least time consuming (mean 5.4 ± 1.8 minutes) [26] (Table 1). However, considering that ILD is usually diffuse, a more comprehensive and careful assessment may be more accurate, especially for screening purposes.

**Table 1:** Four different LUS methods to assess B-lines in SSc patients

	Anatomical line	LUS by Gargani et al. [23]		Comprehensive LUS [25]		Simplified LUS [25]		Modified LUS [26]	
		Right	Left	Right	Left	Right	Left	Right	Left
Anterior	Parasternal	2nd–5th ICS	2nd–4th ICS	2nd–5th ICS	2nd–4th ICS	2nd ICS	2nd ICS		
	Mid-clavicular	2nd–5th ICS	2nd–4th ICS	2nd–5th ICS	2nd–4th ICS	4th ICS	4th ICS	4th ICS	4th ICS
Lateral	Anterior axillary	2nd–5th ICS	2nd–4th ICS	2nd–5th ICS	2nd–4th ICS	4th ICS	4th ICS	4th ICS	4th ICS
	Mid-axillary	2nd–5th ICS	2nd–4th ICS	2nd–5th ICS	2nd–4th ICS	4th ICS	4th ICS	4th ICS	4th ICS
	Posterior axillary	2nd–10th ICS	2nd–10th ICS	7th–8th ICS	7th–8th ICS	8th ICS	8th ICS	8th ICS	8th ICS
Posterior	Sub-scapular	7th–10th ICS	7th–10th ICS	7th–8th ICS	7th–8th ICS	8th ICS	8th ICS	8th ICS	8th ICS
	Paravertebral	2nd–10th ICS	2nd–10th ICS	2nd–8th ICS	2nd–8th ICS	8th ICS	8th ICS		
Total scanning sites		72 ScS		50 ScS		14 ScS		10 ScS	

*LUS* lung ultrasound, *SSc* systemic sclerosis, *ICS* inter-costal space, *ScS* scanning sites

Face validity of LUS has been generally accepted as exemplified by its use when examining SSc-ILD. Construct validity requires correlating LUS with other measures of the same type or reflecting the same pathology, as well as discriminating B-lines for measures of negatively associated aspects of disease. These are called convergent and divergent correlations. These studies are often done using HRCT, thus examining criterion validity at the same time (assuming HRCT is a gold standard for ILD in SSc). Several HRCT scoring methods have been used to characterize and quantify the disease; the Warrick score, a semi-quantitative assessment combining severity and extent of disease, has been applied preferentially [27].

In the study by Gargani et al. [23], the presence of B-lines was observed in 51% of SSc patients, with significantly higher values in the diffuse than in the limited form (73 ± 66 vs 21 ± 35; *p* < 0.05). A statistically significant positive linear correlation was found between B-lines and the Warrick score (*r* = 0.72; *p* < 0.001), and between B-lines and values of diffusing capacity for carbon monoxide (DLCO) (*r* = –0.6; *p* < 0.05). The intra-observer and inter-observer variability of B-line assessment were derived from a previous study by the same group as 5.1% and 7.4% respectively [4].

Tardella et al. [28] also reported a significant linear correlation between the number of B-lines and HRCT score (*p* < 0.001; correlation coefficient ρ = 0.875) and between B-lines and DLCO (*p* = 0.014) in 34 CTD patients (including 26 SSc patients). Inter-observer assessment showed very good agreement (weighted κ value between 0.846 and 0.969, and overall agreement between 92% and 97%). Another study of 58 consecutive SSc patients (including 32 patients with very early SSc) showed a concordance rate of 0.83 between B-lines and HRCT for the assessment of ILD [29]. LUS diagnostic sensitivity and specificity were 100% and 55% respectively, and the negative predictive value (NPV) and positive predictive value (PPV) were 100% and 78% (with a higher cut-off point of ≥ 20 total B-lines, sensitivity was instead 83% and specificity was 96%). The authors also found that patients with ground glass opacity (GGO) by HRCT had a higher total B-line score than those without GGO. Receiver operating characteristic curve (ROC) analysis confirmed the analytical relationship between number of B-lines and the presence of ILD at HRCT (AUC = 0.94, 95% CI 0.89–0.99, *p* < 0.0001). Given the very high sensitivity and negative predictive value, this study proposes B-lines as a screening tool of ILD in SSc patients, to guide further investigation with HRCT. Buda et al. [30] also observed numerous B-lines with a “white lung” pattern to be associated with GGO (*p* < 0.0001), and the sensitivity and specificity were respectively 95% and 99%. In another study comparing 25 SSc patients to 40 healthy controls, all SSc patients with CT signs of ILD (44%) showed B-lines versus only 7% of healthy controls (*p* < 0.001) [31]. Pleural irregularities (PI, defined as the loss of the normal hyperechoic linear pleural contour plus thickening) were also described in this study, although their anatomic correlations and validity are still debated [1, 32]. LUS findings matched the findings on HRCT. Patients with ILD had a higher number of B-lines and higher pleural scores compared with those without radiographic ILD. A similar outcome was observed in a small heterogeneous group of CTD patients (including 25 RA patients, 14 SSc patients, and 6 SLE patients). B-lines were detected in 100% and 12% of patients with or without HRCT-defined ILD, respectively [33]. Subpleural nodes and pleural thickness > 3 mm were observed in 55% and 95% of ILD patients compared to 17% and 12.5% of patients without ILD. In another study of 16 SSc and 21 anti-synthetase syndrome (ASS) patients, PI again showed a high accuracy for detecting radiological ILD [32]. Another study in 175 SSc patients reported that pleural line thickness and subpleural nodules had a good concordance with HRCT patterns indicating pulmonary fibrosis severity, and were able to detect signs of initial pulmonary fibrosis prior to the onset of respiratory symptoms [34].

Several papers have highlighted the correlation between LUS signs with some clinical features. The relationships between B-lines, PFTs, and clinical variables were evaluated in 39 SSc patients [24]. This study confirmed previous data from Gargani et al. [23], showing that the B-line score had a negative correlation with DLCO (*r* = – 0.63, *p* < 0.0001). The number of B-lines increased as capillaroscopic damage in the fingers increased (*p* < 0.01). In addition, the B-line score was significantly higher in patients with diffuse cutaneous SSc (dcSSc) than in those with limited cutaneous SSc (lcSSc) (*p* < 0.05), and in patients with digital ulcer history than in those without digital ulcer history (*p* < 0.01). The Medsger scleroderma disease severity scale also had a significant correlation with B-lines (*r* = 0.80, *p* < 0.01). No significant association was observed with disease duration, mRSS, or European Scleroderma Study Group activity index.

## B-lines in rheumatoid arthritis-associated interstitial lung disease

There are two studies examining LUS in RA patients [35, 36]. A prospective study of 64 RA outpatients without clinical pulmonary symptoms revealed that 28% of patients had B-lines or pleural nodules [35]. In 89% of LUS-positive patients, HRCT scans showed signs of ILD. This established criterion validity for LUS with the “usual” assumption that HRCT represents the gold standard. LUS also showed sporadic abnormalities in 7% of the healthy controls. Agreement between LUS and HRCT yielded a sensitivity of 97.1% and a specificity of 97.3%. The predefined criteria yielded a PPV of 94.3% and NPV of 98.6%. These encouraging results will need to be corroborated in larger studies.

Another study has compared two different US devices to detect B-lines in a small cohort of RA patients. Both standard (using a 2–5 MHz convex probe) and pocket-size US (PS-US, using a 1.7–3.8 MHz phased array transducer) devices were used to examine lungs characterized by radiological ILD in 39 RA patients [36]. A B-line score > 10 identified a positive examination. The study found that sensitivity and specificity of standard LUS and PS-LUS vs HRCT were 92% and 56%, and 89% and 50%, respectively. The κ coefficient between the two methods was 0.78, indicating that PS-US devices can provide a diagnostic accuracy similar to higher-level devices.

These data may help define the utility of LUS in RA, but other crucial aspects of this device have not yet been proven valid in RA, including its reproducibility, reliability, and applicability to a wide range of patients. Responsiveness and discrimination are unknown. Hopefully, much of the work done in SSc can be applied here, but caution is justified.

## B-lines in Sjögren’s syndrome-associated interstitial lung disease

More recently, it has been demonstrated that B-lines are well correlated to the HRCT sign of pulmonary fibrosis in Sjögren’s syndrome (SS) patients [37]. Thirteen SS patients were evaluated by LUS and chest HRCT, independently performed within 6 months. B-lines were evaluated on eight thoracic zones. A zone was considered positive if at least three B-lines were identified in a single ICS. LUS showed a sensitivity of 1 (95% CI 0.398–1.0), a specificity of 0.89 (95% CI 0.518–0.997), and a positive probability reason of 9 (95% CI 7.1–11.3) to detect ILD. LUS had good correlation with HRCT (*r* = 0.84, *p* < 0.001) and high accuracy to diagnose ILD (AUC = – 0.94, 95% CI 0.81–1.0, *p* = 0.014). Although the data are promising, the positive evidence relies on a very small number of patients. Another limitation is the long time frame between LUS and HRCT, with potential bias linked to changes in lung parenchyma over time.

## B-lines in anti-synthetase syndrome-associated interstitial lung disease

The correlation of B-lines with HRCT was studied in 22 ASS patients [38]. B-lines were analysed semi-quantitatively (a maximum of B-lines, calculated as the percentage of positive sonographic points, divided by the number of sonographic points studied per patient) and were most often found in the lower posterior and upper anterior areas. The κ values for intra-observer and inter-observer reliability were 0.83 and 0.76, respectively. The median HRCT Warrick score was 15 (Q1–Q3 13–22), with GGO affecting the largest number of segments (median 10 (Q1–Q3 6–12)), followed by irregular pleural margins (median 6 (Q1–Q3 4–110)), and septal/subpleural lines (median 6 (Q1–Q3 0–10)). No significant correlation was found between the percentage of B-lines and the overall Warrick score. When correlation with the different components of Warrick’s score was analysed, only the number of HRCT segments showing GGO was related to the percentage of B-lines (ρ = 0.5, *p* = 0.02). In a subsequent study from the same group on patients with both SSc and ASS, PI showed a better performance than B-lines to detect ILD [32]. These results are partially not consistent with previous studies; a possible explanation being the different scoring systems. Further research is needed to better understand the role of LUS in ASS, and especially to evaluate the role of PI in these conditions.

**Fig. 1:**
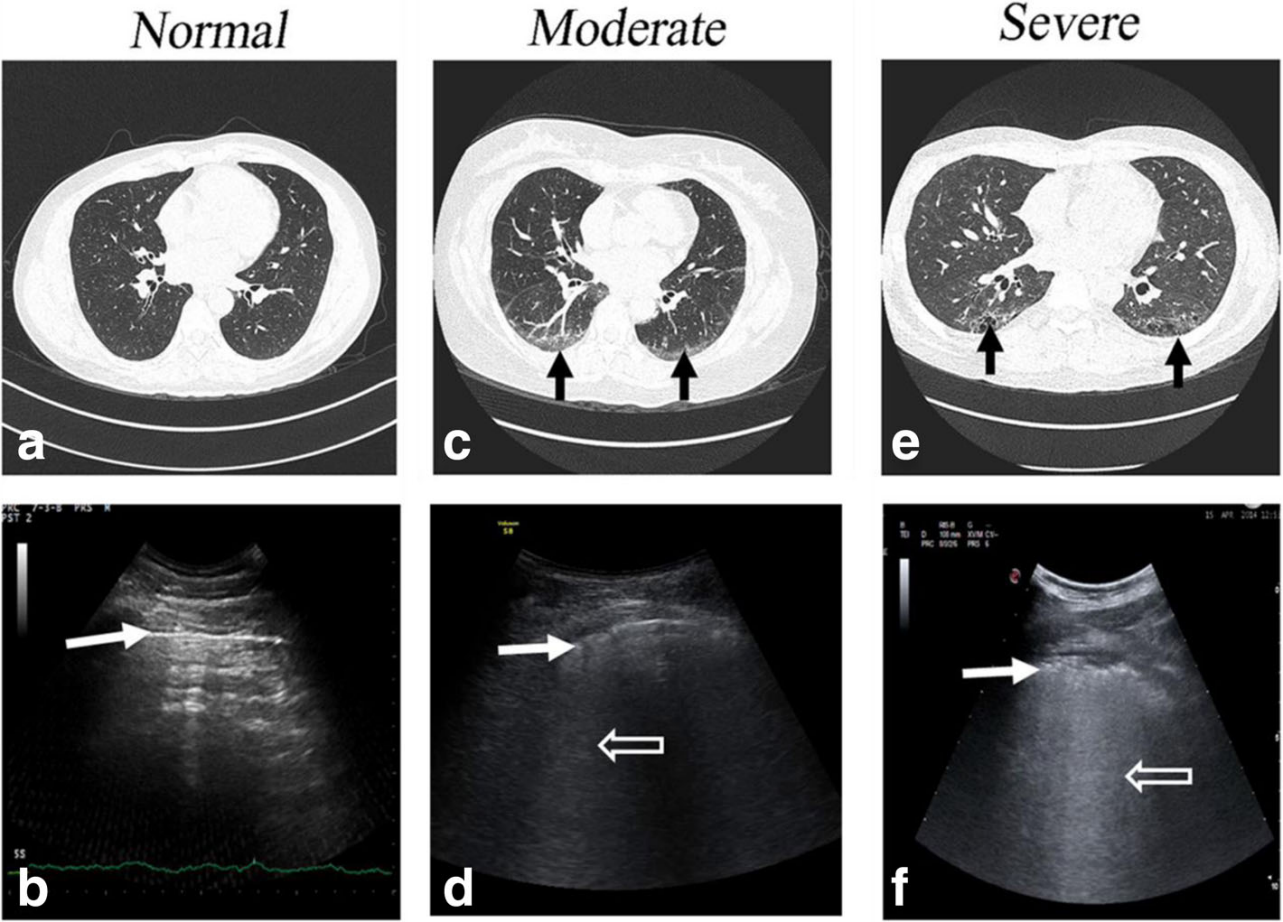
Different HRCT and LUS patterns. **a** HRCT: normal. **b** LUS: normal pleural line (white arrow). No B-lines visible. **c** HRCT: ground-glass opacity (black arrow). **d** LUS: blurred and irregular pleural line (white arrow) and multiple B-lines (white empty arrow). **e** HRCT: honey-combing (black arrow). **f** LUS: blurred and irregular pleural line (white arrow) and multiple B-lines (“white lung”, white empty arrow). HRCT high-resolution computed tomography, LUS lung ultrasound

All of the included studies are depicted in Table 2. Different HRCT and LUS patterns of absent, moderate, and severe fibrotic involvement are shown in Fig. 1.

**Table 2:** Overview of included studies

Study	Content	Construct	Criteria	Feasibility	Reliability	Discrimination	Responsiveness
Gargani et al. [23]	33 SSc, including 10 dcSSc, and 23 lcSSc	LUS HRCT (gold standard) PFT	72 ScS Total BN > 10 was defined positive WS	100%	Intra-observer and inter-observer variability respectively 5.1% and 7.4%	Total BN correlated with WS (*r* = 0.72; *p* < 0.001) and DLCO (*r* = – 0.6, *p* < 0.05); BN higher in dcSSc than in lcSSc (73 ± 66 vs 21 ± 35; *p* < 0.05)	N/A
Gutierrez et al. [25]	28 SSc, 2 SS, 2 DM, 2 ASS, 1UCTD, and 1 MCTD	LUS HRCT (gold standard)	50 ScS (comprehensive method) 14 ScS (simplified method); comprehensive BS was defined 0 = normal (<10 BN); 1 = mild (11–20 BN); 2 = moderate (21–50 BN), and 3 = marked (>50 BN). Simplified BS was defined 0 = normal (<5 BN); 1 = mild (6–15 BN); 2 = moderate (16–30 BN), and 3 = marked (>30 BN) WS	Simplified method required less time than the comprehensive (8.6 ± 1.4*vs* 23.3 ± 4.5 minutes, *p* < 0.001)	κ values for inter-observer reliability of comprehensive method 0.85–0.98. κ values for inter-observer agreement of simplified method 0.77–0.89 and for intra-observer 0.85–0.89	BS of two methods correlated to WS (*p* = 0.0006), and simplified score also correlated to comprehensive method (*p* = 0.0001)	N/A
Barskova et al. [29]	58 SSc, including 32 VEDOSS	LUS HRCT (gold standard)	72 ScS BN ≥ 3 was found in at least two adjacent scanning sites or when total BN > 5	100%	Intra-observer and inter-observer variability respectively 5.1% and 7.4%	Total BN significantly higher in SSc + ILD (57 ± 53 vs 9 ± 9, *p* < 0.0001), and with GGO (63 ± 47 vs 33 ± 40, *p* < 0.05) Sensitivity and specificity respectively 100% and 55%; NPV and PPV 100% and 78% respectively	N/A
Tardella et al. [28]	26 SSc, 2 SS, 1 UCTD, 2 ASS, 2 DM, and 1 MCTD	LUS HRCT (gold standard) PFT	50 ScS Grading as comprehensive assessment (Gutierrez et al. [25])	Yes	Overall agreement of inter-observer 92–97%; weighted κ value 0.85–0.98	BS correlated with WS (*p* < 0.001; CC ρ = 0.875), and DLCO(*p* = 0.014)	N/A
Moazedi-Fuerst et al. [31]	25 SSc and 40 healthy controls	LUS HRCT (only for patients)	18 ScS Positive area for B lines = ScS with predominant B lines; positive area for PI = predominant PLT > 2.8 mm BS: 0 = no positive areas; 1 = 1–5 positive areas; 2 = > 5 positive areas. Idem for PI	N/A	N/A	SSc + ILD had a BS of 2 in 55% and 1 in 45%; SSc – ILD had a BS of 2 in 5% and BS of 1 in 30%; SSc + ILD had a PI of 2 in 23% and 1 in 78%; SSc – ILD had a negative PI	N/A
Pinal Fernández et al. [38]	21 ASS	LUS HRCT (gold standard)	72 ScS Percentage of positive B-lines calculated (dividing the positive points by studied points) WS	N/A	κ value of intra-observer and inter-observer 0.83 and 0.76	BS no correlated with WS (CC = 0.135, *p* = 0.559); BS correlated with GGO (ρ = 0.502, *p* = 0.02)	N/A
Cogliati et al. [36]	39 RA	LUS (standard and PS-USD) HRCT (gold standard) PFT	72 ScS BN > 10 identified positive WS	Yes	*r*-value for inter-observer variability 0.96; κ coefficient of two devices 0.78	BS correlated with WS (*r* = 0.806). Sensitivity and specificity of standard LUS vs HRCT 92% and 56%, and PS-USD vs HRCT 89% and 50%	N/A
Moazedi-Fuerst et al. [35]	64 RA and 40 healthy controls	LUS HRCT (gold standard, only for patients)	18 ScS Grading as previous report (2012)	Yes	κ value of inter-observer 0.92	Sensitivity and specificity of LUS respectively 97.1% and 97.3%; PPV and NPV 94.3% and 98.6% respectively (*p* < 0.001)	N/A
Mohammadi et al. [26]	70 SSc	LUS (modified TTUS) HRCT (gold standard)	10 ScS BS: 0 = normal (≤5 BN); 1 = mild (6–15 BN); 2 = moderate (16–30 BN); 3 = severe (>30 BN) WS	Yes	κ value of intra-observer reliability 0.838. Agreement between LUS and HRCT 0.553 (*p* < 0.001)	BS correlated with WS (SCC = 0.695, *p* < 0.001); sensitivity and specificity respectively 73.5% and 88.23%; PPV and NPV 95.12% and 51.72% respectively	N/A
Gigante et al. [24]	39 SSc, including 24 dcSSc and 15 lcSSc	LUS HRCT (gold standard) PFT NVC	BN ≥ 3 was found in at least two adjacent scanning sites or when a total BN > 5 WS	N/A	Intra-observer variability 3.8%	BN correlated with WS (*r* = 0.81, *p* < 0.0001), DLCO (*r* = – 0.63, *p* < 0.0001), and DSS (*r* = 0.8, *p* < 0.01)	N/A
Moazedi-Fuerst et al. [33]	25 RA, 14 SSc, 6 SLE, and 40 healthy controls	LUS HRCT (gold standard, only for patients)	18 ScS BN > 2 and PLT > 3 mm in any scanned area regarded as abnormal. Grading as previous report (2012)	Yes	N/A	Sensitivity and specificity of LUS respectively 86.9% and 100%; PPV and NPV 100% and 88% respectively	N/A
Pinal-Fernandez et al. [32]	16 SSc and 21 ASS	LUS HRCT (gold standard) PFT	72 ScS Percentage of positive BN and PI calculated (dividing the positive points by studied points) WS and Wells scores	N/A	N/A	PI correlated with WS both in SSc (*r* = 0.6, *p* = 0.01) and ASS (*r* = 0.6, *p* = 0.005), higher performance than BS (*p* = 0.01). PI also correlated with Wells score (*r* = 0.7, *p* < 0.001) and with DLCO (*r* = – 0.5, *p* = 0.05) in SSc, and high diagnostic value for detecting ILD (AUC = 0.85, 95% CI 0.64–1) and classified limited and extensive (AUC = 0.81, 95% CI 0.57–1)	N/A
Buda et al. [30]	52 ILD (including 30 CTD and 16 IP) and 50 healthy controls	LUS HRCT (gold standard)	BN classified into three types: single (≤3 per one scan), numerous (≥4), and white lung. PI was described ragged, fragmentary, thickness (≥2 mm), and blurred WS	N/A	N/A	Sensitivity and specificity of white lung to GGO.95% and 99%; blurred pleural line to honeycombing 59% and 82% (p < 0.005); numerous B lines correlated with blurred pleural line (*p*<0.001)	N/A
Sperandeo et al. [34]	175 SSc	LUS HRCT (gold standard)	BN > 3; PLT > 3.0 mm; HRCT pattern classified: no fibrosis, reticular, reticular-nodular, and honeycombing + reticular-nodular pattern	N/A	κ value of inter-observer and intra-observer 0.6–0.8	Sensitivity and specificity of PLT (>3.0 to ≤ 5.0) to reticular pattern 80% and 99% (AUC = 0.95); PLT (>3.5) to reticular nodular and honeycombing 74.3% and 99% (AUC = 0.99); PLT (>5) to honeycombing 90.1% and 99% (AUC = 0.99) Sensitivity and specificity of BS to all kinds of fibrosis 0.94 and 0.95	N/A
Vasco et al. [37]	13 SS	LUS HRCT (gold standard)	8ScS BN ≥ 3 in a single ScS	N/A	κ value of intra-rater 1	BS correlated with HRCT (*r* = 0.84, *p* < 0.001); sensitivity and specificity respectively 100% and 8﻿9%	N/A

*ASS* anti-synthetase syndrome, *AUC* area under curve, *BN* B-line number, *BS* B-line score, *CC* correlation coefficient, *CI* confidence interval, *CTD* connective tissue disease, *dcSSc* diffuse cutaneous SSc, *DLCO* diffusion capacity for carbon monoxide, *DM* dermatomyositis, *DSS* disease severity scale, *GGO* ground glass opacity, *HRCT* high-resolution computed tomography, *ILD* interstitial lung disease, *IP* idiopathic pneumonia, *lcSSc* limited cutaneous SSc, *LUS* lung ultrasound, *MCTD* mixed connective tissue disease, *N/A* not applicable, *NPV* negative predictive value, *NVC* nailfold video capillaroscopy, *PFT* pulmonary function test, *PI* pleural irregularity, *PLT* pleural line thickening, *PPV* positive predictive value, *PS-USD* pocket size ultrasound device, *RA* rheumatoid arthritis, *ScS* scanning sites, *SLE* systemic lupus erythematosus, *SCC* Spearman’s correlation coefficient, *SS* Sjögren’s syndrome, *SSc* systemic sclerosis, *SSc + ILD* SSc with ILD, *SSc – ILD* SSc without ILD, *TTUS* transthoracic ultrasound, *UCTD* undifferentiated connective tissue disease, *VEDOSS* very early diagnosis of systemic sclerosis, *WS* Warrick score

## Limitations

The studies outlined showed promising results in selected diseases and circumstances. LUS is almost fully validated in SSc (lacking only discrimination) and SS (very small number patients), and is incompletely validated in RA and ASS. Complete validation is lacking in all of these diseases, making the use of LUS somewhat preliminary, despite being very promising and attractive.

B–lines can have various aetiologies (e.g. interstitial oedema, interstitial fibrosis) [8, 39, 40], and differentiating them in clinical practice can sometimes be difficult. In particular, in CTD-ILD B-lines alone cannot differentiate the early inflammatory phase from the chronic fibrotic phase [41, 42], which could potentially lead to some misclassification bias, although this differentiation is also often not so easy by HRCT. The role of pleural abnormalities, which seems promising in completing the information provided by B-lines, warrants more specific studies, and it is debatable whether measuring the sonographic pleural line could be meaningful in normal and pathologic conditions.

Up to now, no method to objectively score B-lines has been provided (although computer algorithms are under investigation) [43, 44]. In the future, this area should be a priority for research.

Furthermore, the majority of studies focused on B-lines as a diagnostic tool in ILD. No data are yet available on B-lines for follow-up in rheumatic disease patients, nor on the accuracy of this method to assess the eventual response to therapy (only two case reports are available) [45, 46], nor on the correct timing of LUS for diagnosis and follow-up. All studies up to now have included rather small populations from a single centre.

Finally, no data are available on large normal populations to confirm the cut-off points and PPV and NPV for this technique in SSc, RA, and other CTDs [47–50].

## Conclusions

LUS is an attractive and promising technique, which may become an important clinical tool to be integrated with HRCT and PFT in the screening and evaluation of ILD. To date, B-lines are waiting to be validated fully in CTD, and the role and meaning of sonographic pleural irregularities must be more clearly elucidated.

## Abbreviations

ASS: Anti-synthetase syndrome
CTD-ILD: Connective tissue disease-associated interstitial lung disease
dcSSc: Diffuse cutaneous systemic sclerosis
DLCO: Diffusing capacity for carbon monoxide
GGO: Ground glass opacity
HRCT: High-resolution computed tomography
lcSSc: Limited cutaneous systemic sclerosis
LUS: Lung ultrasound
NPV: Negative predictive value
PFT: Pulmonary function test
PI: Pleural irregularities
PPV: Positive predictive value
PS-US: Pocket-size ultrasound
RA: Rheumatoid arthritis
ROC: Receiver operating curve
SLE: Systemic lupus erythematosus
SSc: Systemic sclerosis
US: Ultrasound
